# Genomic Epidemiology Linking Nonendemic Coccidioidomycosis to Travel

**DOI:** 10.3201/eid2901.220771

**Published:** 2023-01

**Authors:** Juan Monroy-Nieto, Lalitha Gade, Kaitlin Benedict, Kizee A. Etienne, Anastasia P. Litvintseva, Jolene R. Bowers, David M. Engelthaler, Nancy A. Chow

**Affiliations:** Translational Genomics Research Institute, Pathogen and Microbiome Division, Flagstaff, Arizona, USA (J. Monroy-Nieto, J.R. Bowers, D.M. Engelthaler);; Centers for Disease Control and Prevention, Atlanta, Georgia, USA (L. Gade, K. Benedict, K. Etienne, A.P. Litvintseva, N.A. Chow)

**Keywords:** coccidioidomycosis, fungi, respiratory infections, whole-genome sequencing, Coccidioides phylogeography, epidemiology, travel-related illness, airborne infections, genomics

## Abstract

Coccidioidomycosis is a fungal infection endemic to hot, arid regions of the western United States, northern Mexico, and parts of Central and South America. Sporadic cases outside these regions are likely travel-associated; alternatively, an infection could be acquired in as-yet unidentified newly endemic locales. A previous study of cases in nonendemic regions with patient self-reported travel history suggested that infections were acquired during travel to endemic regions. We sequenced 19 *Coccidioides* isolates from patients with known travel histories from that earlier investigation and performed phylogenetic analysis to identify the locations of potential source populations. Our results show that those isolates were phylogenetically linked to *Coccidioides* subpopulations naturally occurring in 1 of the reported travel locales, confirming that these cases were likely acquired during travel to endemic regions. Our findings demonstrate that genomic analysis is a useful tool for investigating travel-related coccidioidomycosis.

*Coccidioides immitis* and *C. posadasii*, the etiologic agents of coccidioidomycosis, also known as Valley fever, are environmental filamentous fungi with distinct geographic ranges in the western United States, northern Mexico, and parts of Central and South America (*1*,*2*). In 2015, *C. immitis* was discovered in Washington, USA (*3*). That discovery highlighted the importance of using molecular detection methods (*3*,*4*) and enhanced efforts to study *Coccidioides* spp. outside traditionally identified endemic areas. Finding that *Coccidioides* might exist outside its previously established endemic regions supports the hypothesis that the geographic range of this pathogen may be changing (*5*). 

In recent years, genomic analyses of this pathogenic genus uncovered strong phylogeographic structure and delineated populations associated with specific geographic regions, findings that expand previous work based on immunological studies (*6*) and molecular studies using traditional, less-discerning methods (*7*). *C. immitis* is found primarily in California and Washington, *C. posadasii* in Arizona, Texas, Mexico, and Central and South America (*8*). Within species, population structure has been characterized, separating the Washington isolates from other *C. immitis* strains (*3*,*9*) and dividing *C. posadasii* into several differentiated phylogeographic clades (*10*), including the Arizona and Texas/Mexico/South America (TX/MX/SA) clades, and a more recently delimited Guatemala/Venezuela clade (*11*). Although no phenotypic distinction between these groups has been associated with disease outcome, the genetic differences and population structures among these clades represent considerable assets for molecular epidemiology and enable tracking of the origins of infections. 

*Coccidioides* spp. can infect several species of mammals, including humans. Both immunocompromised and immunocompetent persons can develop coccidioidomycosis by inhaling airborne propagules from disturbed soil (*2*). In human hosts, symptoms range from inconsequential to self-limited and often protracted respiratory illness to chronic pulmonary disorders and, in rare cases, disseminated systemic infections (*12*,*13*). In the United States, >10,000 new cases/year have been reported to public health authorities in recent years, mainly in Arizona and California (*13*,*14*). Those states are also thought to be the source of most travel-related infections, as confirmed by the results of enhanced surveillance from 2016 describing clinical and epidemiologic characteristics of reported cases from 14 coccidioidomycosis nonendemic states (*12*). In that study, most patients had either traveled to coccidioidomycosis-endemic regions during the 4 months before symptom onset or visited or previously resided in an endemic region at some point during their lifetimes. Previous studies (*3*,*15*) have shown that isolates can be traced to their place of origin by applying current understanding of *Coccidioides* phylogeography to the increasing number of publicly available genomes. We determined to further investigate the isolates from cases described in the 2016 enhanced surveillance by using genomic epidemiology to empirically evaluate the original findings and identify likely origins of coccidioidomycosis cases in nonendemic regions. 

## Methods 

### Sample Descriptions

We included 174 genomes (104 *C. posadasii* and 70 *C. immitis*) in this study (Appendix 1). For the study, we processed 72 (41%) at the reference laboratory of the Mycotic Diseases Branch, Division of Foodborne, Waterborne, and Environmental Diseases, National Center for Emerging and Zoonotic Infectious Diseases, Centers for Disease Control and Prevention; 53 of the isolates were collected for routine fungal reference testing and 19 (16 *C. posadasii*, 3 *C. immitis*) as part of the enhanced surveillance study (*12*). For the routinely collected set, we received limited metadata, such as geographic region of collection, location where the infection was acquired, and patient travel history. The enhanced survey set included this same information, collected from interviews as described in the original publication (*12*); in brief, patients were asked about history of travel to any endemic area within 4 months before disease onset. Some samples sent from health departments in different jurisdictions we subsequently determined to be duplicates of multiple isolates (B17635, B14131, B15145) from the same patient. Those genomes were presented as unique clonal leaves to illustrate that they corresponded to multiple physical specimens and genome libraries of the same strain. Additional whole-genome sequences downloaded from the National Center for Biotechnology Information (NCBI) Sequence Read Archive (SRA) amounted to 102 (59% of the total) additional public samples included in our analyses; those sequences had been deposited under BioProjects PRJNA245906 (*3*), PRJNA472461 (*9*), PRJNA274372 (*10*), PRJNA438145 (*11*), and PRJNA46299 (*16*). 

For the complete dataset, data for 144/174 (83%) isolates were from clinical cases of coccidioidomycosis, 11 (6%) environmental samples from previous studies (*3*,*10*) or reference testing, and 1 veterinary sample; 18 (10%) did not have this information available. Patient location, defined as where the *Coccidioides* sample was collected and its associated case reported, was known for 172 (99%) of the samples. Most of the variety of regional locations were in US states: 43 (25%) in Washington, 32 (18%) in Arizona, 29 (17%) in Oregon, 14 (8%) in California, and 11 (6%) in Michigan. An additional 10 (6%) locations were in Mexico, 7 (4%) in Venezuela, and 28 (16%) in other regions. We collected all 19 patient samples in the enhanced surveillance effort (*12*) from nonendemic areas. 

### DNA Extraction and Genome Sequencing

We grew all isolates on brain-heart infusion agar at 25°C for 10 days, extracted high molecular genomic DNA using the DNeasy Blood and Tissue kit (QIAGEN, https://www.qiagen.com) according to manufacturer recommendations, and confirmed isolates by sequencing the internal transcribed spacer 2 region of the rDNA. We stored genomic DNA at −20°C for future use. We constructed and barcoded genomic libraries using NEBNext Ultra DNA Library Prep kit for Illumina (New England Biolabs, https://www.neb.com), following manufacturer instructions. We sequenced libraries on either the Illumina HiSeq 2500 platform (https://www.illumina.com) using the HiSeq Rapid SBS Kit v2 500 cycles or the Illumina MiSeq platform using the MiSeq Reagent Kit v2 500 cycles, generating paired reads 250 bp in length. We deposited read data to the National Center for Biotechnology Information Sequence Read Archive, bundling the read files under Bioproject PRJNA808058. 

### Phylogenomic Analyses

Using the genomic sequence data from the selected isolates and publicly available genomes (Appendix 1), high-confidence single-nucleotide polymorphisms (SNPs) were identified with the Northern Arizona SNP Pipeline (NASP) v1.2.0, a genome analysis tool (*17*). For read data, we performed quality trimming for flanking regions with an average quality score <20 using BBduk version 38.26 from BBtools (https://www.sourceforge.net/projects/bbmap). Subsequently, in NASP, we used Burrows-Wheeler aligner (https://bio-bwa.sourceforge.net) to align reads to reference genomes: RMSCC3488 (GCA_000150055.1) for *C. posadasii* and RS (GCA_000149335.2) for *C. immitis* (*18*). We identified SNPs with GATK’s UnifiedGenotyper (*3**,**7**,**19*). We analyzed previously assembled genomes as described elsewhere (*17*). NASP filtered out SNPs where >1 sample had <10× coverage or <90% concordance among the aligned reads and loci masked in the reference duplicated using MUMmer (*20*). We used the remaining high-certainty genomic SNPs to reconstruct phylogenies using maximum-likelihood inference using the best scoring fit models, transversion plus ascertainment bias correction plus R5 for *C. posadasii* and transition model 3 plus ascertainment bias correction plus R5 for *C. immitis*, after conducting a full search of all available models in IQTREE (*21*) with 1,000 ultrafast bootstraps. We rooted trees by the next basal branch using the genome for *Uncinocarpus reesii* 1704 (GCA_000003515.2) as an outgroup. We used the ggtree plotting library (*22*) to generate tree figures. 

## Results 

**Figure 1:**
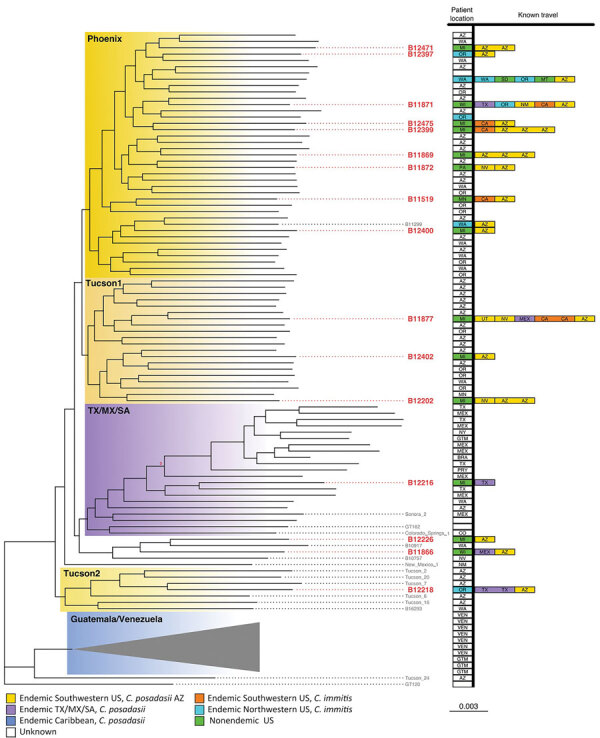
Summarized maximum-likelihood phylogenetic tree for *Coccidioides posadasii* isolates from study of genomic epidemiology linking nonendemic coccidioidomycosis to travel and reference isolates. Each recognized phylogeographic clade is highlighted with a colored gradient labeled in its top right corner. Samples with travel history are presented with locations of isolation and all known patient travel, with colors for the geographic regions. Red bold tip labels indicate samples sequenced for this study; other samples are included when including travel history or otherwise mentioned in the main text.

**Figure 2:**
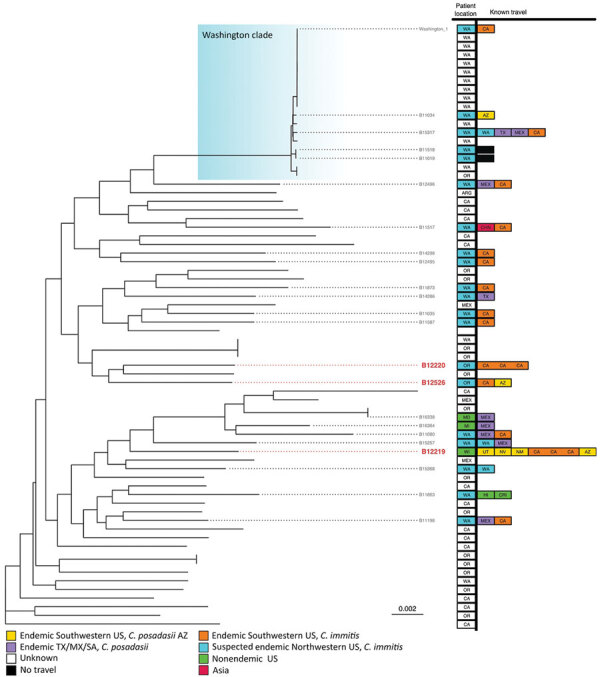
Summarized maximum-likelihood phylogenetic tree for *Coccidioides immitis* isolates from study of genomic epidemiology linking nonendemic coccidioidomycosis to travel and reference isolates. Each recognized phylogeographic clade is highlighted with a colored gradient labeled in its top right corner. Samples with travel history are presented with locations of isolation and all known patient travel, with colors for the geographic regions. Red bold tip labels indicate samples sequenced for this study; other samples are included when including travel history or otherwise mentioned in the main text.

We performed separate phylogenetic analyses for samples of each species. Most (n = 99, 95%) *C. posadasii* samples clustered with 5 clades: Phoenix, Tucson 1, Tucson 2, TX/MX/SA, and Guatemala/Venezuela (Figure 1). *C. immitis* samples clustered mainly with the Washington clade (Figure 2). We also identified 7 geographic regions to categorize travel history locations: endemic southwestern United States (*C. immitis* and *C. posadasii*­), endemic TX/MX/SA, suspected endemic northwestern United States (*C. immitis* and *C. posadasii*­), endemic Caribbean, nonendemic United States, and Asia. 

Of the 19 *Coccidioides* spp. isolates from travel-related cases from the enhanced surveillance study, 16 (84%) were *C. posadasii* and 3 (16%) were *C. immitis*. Of the 16 *C. posadasii* isolates, 13 (81%) clustered with isolates from the endemic southwestern United States region, consistent with reported patient travel to Arizona, and 1 with the TX/MX/SA group from a patient who had traveled to Texas. Isolates from the remaining 2 travel-related cases clustered with an outgroup to the TX/MX/SA clade not previously reconstructed, whose southwest United States geographic distribution has not been resolved. That outgroup included isolates collected from cases in Nevada and New Mexico, US states with some recognized endemicity, and a single isolate from a case with unknown history in Washington. Isolate B11866 was collected in Wisconsin from a patient who reported having traveled to Mexico or Arizona. Isolate B12226 was collected in Michigan, and the patient reported travel to only 1 endemic area, within the Phoenix metropolitan area in Arizona. 

The 3 travel-related cases caused by *C. immitis* all had samples nested within the clades linked to California, another geographic region reported by patients in their travel histories (Figure 2). Two cases had isolates, B12220 and B12526, that clustered with 28 isolates collected in the suspected endemic northwestern United States region but nested within the Californian clade (Figure 2), which was consistent with the case-patients’ reported travel histories. The isolate associated with the third *C. immitis* genome, which also clustered within the California clade, was recovered in Wisconsin from a patient who had traveled to several endemic regions throughout the southwestern United States.

## Discussion 

*Coccidioides* spp. fungi have long been thought to be limited to the southwestern United States, northern Mexico, and parts of Central and South America. Endemicity has been determined by case epidemiology, population-based skin test surveys, and, of note, from direct environmental detection (*23*). However, detection of *Coccidioides* spp. in the states of Utah in 2014 (*24*) and Washington in 2015 (*4*), as well as genomic analyses demonstrating *C. immitis* in Washington as a distinct clade (*9*,*10*), challenged our knowledge of this pathogen’s true geographic distribution. Previous public health enhanced surveillance for coccidioidomycosis in nonendemic states showed that most cases were more likely attributed to travel than to local acquisition (*12*). The phylogeographic analysis of genome sequences from our epidemiology study corroborated those findings. Most of these isolates were from specific populations of *Coccidioides* spp. in regions known to be endemic to which case-patients had traveled. 

We had detailed travel histories for all patients isolates from the 19 travel-related cases. Of 2 patients diagnosed in Michigan, one, associated with isolate B12399 (*C. posadasii* Phoenix clade), had not traveled to any known endemic area in the previous 10 years, and the other, associated with B11877 (*C. posadasii* Tucson 1 clade), had not traveled to any endemic area for 4 years, time periods longer than those usually queried on travel histories used to determine the risk of coccidioidomycosis. Those 2 cases from our study exemplify how travel to endemic areas might remain a risk factor for the disease even after several years. Therefore, healthcare providers should consider patients’ lifetime travel histories when diagnosing illnesses, especially among patients potentially immunocompromised by coexisting medical conditions. 

Genomic analysis offers an opportunity to address several unanswered epidemiologic questions about coccidioidomycosis. Clarifying the biogeographic distribution of *Coccidioides* spp. (*8*) was a necessary outcome of these studies. A case associated with clinical isolate B12226 was reported in Michigan, in which the case-patient had a travel history to Arizona. However, unexpectedly, this genome clustered in the tree with a set of leaves neighboring the TX/MX/SA clade. Contrary to what would be expected based on reported travel history, this adjacent group is not part of the Arizona subpopulations. The topology and bootstrapping support for this neighboring Arizona clade suggest that it is a sibling subpopulation to the TX/MX/SA clade that has remained undersampled and thus not reconstructed until now. Bootstrap values in unabridged phylogenic trees (Appendix 2) show that the geographic origin of the Arizona clade remains unclear; the provenance of other clinical isolates in that branch includes New Mexico and Nevada, which obfuscates its probable geographic distribution. Sequencing both isolates from local patients with no travel history or environmental isolates belonging to this clade could help delineate the geographic borders of this subpopulation of *C. posadasii*. This gap in our understanding could potentially hinder the methodology that enabled this study because isolates in this group could not be traced unambiguously to their expected origins. This technique will most likely improve as more *Coccidioides* genomes are sequenced and population genomic efforts continue. 

Conducting genomic sequencing more routinely could shed light on important clinical questions such as how long the infection can remain dormant before it reactivates and causes disease. Clinical cases of coccidioidomycosis have often been thought to represent relapses of previous infections; however, lack of documentation and difficulty tracking individual patients hindered this determination. Some reports of relapse indicate initial infections dating back several decades (*25*) and many patients in the 2016 enhanced surveillance study had a self-reported previous history of the illness (*12*). By procuring isolates during different instances of illness in the same patient, whole-genome sequencing could provide definitive evidence of the origin of the infection in each case, helping to resolve this longstanding clinical question. 

More widespread phylogenetic typing platforms would greatly benefit epidemiologic efforts to understand *Coccidioides* spp. from a public health perspective. Increasingly available, whole-genome sequencing provides a reliable method for assigning pathogens to phylogenetic clades, enabling detection of the causes of outbreaks with unparalleled resolution. For our study, whole-genome sequencing provided information needed to resolve additional *Coccidioides* spp. population structure and enable further research into its pathogenicity. 

Currently, a handful of isolates have remained challenging to assign to specific clades because of long terminal branches and low bootstrap support for leaf nodes in phylogenetic trees of *Coccidioides* genomes. Different analyses have not consistently placed *C. posadasii* isolates Sonora 2, Tucson 2, and Tucson 6 within the same clades (*10*,*26*). The long branches that separate any 2 genomes may result in long-branch attraction, making reconstruction of phylogenies highly contingent on the sampling selection and phylogenetic reconstruction algorithm and resulting in errors in tree topology (*27*) or uncertainty in assigning isolates (e.g., GT162, Colorado_springs_1). Our reconstruction of the *C. posadasii* species tree illustrates an ancestral lineage, here labeled Tucson 2, bearing similarities to the clade AZ clade I (*11*,*26*), but the Tucson and Arizona clades differ in their topological relations to other clades and the member isolates they contain. Those distinctions might become visible because of the increased genetic context provided by whole-genome sequencing to resolve phylogenetic relationships. Support for this lineage was present in previously published studies (*10*,*11*) that showed several of the isolates in this branch having admixture compositions different from those of the rest of the AZ and the TX/MX/SA clades. 

The retrospective nature of this study limited our ability to review charts to acquire additional information of interest, including evidence of possible long-term infections. Our reliance on patients’ missing or self-reported, possibly incomplete, travel histories limited information linked to location, especially for isolates collected outside of enhanced surveillance. Even though some patients included limited travel information, it could not be used with the same certainty as travel information collected systematically. Specifically, most isolates submitted from Oregon lacked patient travel information. Because most Oregon patients in this study were residents of the Portland metropolitan area, which has a humid, temperate climate not known to support endemic *Coccidioides* spp. population, those infections were likely acquired during travel. This conclusion, although based on extrapolation, does not conflict with other conclusions drawn in our study. Other irregularities in collection included 3 instances in which multiple isolates from the same patient were submitted, including 2 cases in which isolates were submitted independently by Oregon and Washington public health laboratories because the patient was diagnosed in one state but resided in the other. As indicated in the methods, we included all genomes that were sequenced separately. Clonal leaves are not expected results for coccidiomycosis cases and should be revised to identify collection irregularities. Such challenges require transversal solutions to capture, collect, and transmit information that might facilitate public health, clinical, and research efforts. 

Despite challenges to reconstructing the population structure of *Coccidioides* spp., we were able to reliably correlate phylogeography and patient travel history in most cases of infection with these fungi. Further isolation and sequencing may better inform epidemiology and improve our understanding of the phylogeography of the *Coccidioides* species and their spatially linked lineages. Our results strengthen previous findings and underscore the importance of travel considerations when studying and diagnosing coccidiomycosis inside and outside known *Coccidioides-*endemic areas. 

Appendix 1 Strain information for genomes used in study of genomic epidemiology linking nonendemic coccidioidomycosis to travel and reference isolates.

Appendix 2 Complete maximum-likelihood phylogenetic trees for *Coccidioimycosis posadasii* and *C. immitis* isolates from study of genomic epidemiology linking nonendemic coccidioidomycosis to travel and reference isolates.
