# Supplementary material for: Genomic Epidemiology Linking Nonendemic Coccidioidomycosis to Travel
Source: Emerg Infect Dis. 2023 Jan;29(1):110–7. doi: 10.3201/eid2901.220771 (PMC9796224; doi:10.3201/eid2901.220771)
Supplement: Appendix 2 — Complete maximum-likelihood phylogenetic trees for Coccidioimycosis posadasii and C. immitis isolates from study of genomic epidemiology linking nonendemic coccidioidomycosis to travel and reference isolates. [file 22-0771-Techapp-s2.pdf]

Article DOI: https://doi.org/10.3201/eid2901.220771

# Genomic Epidemiology Linking Nonendemic Coccidioidomycosis to Travel

## Appendix 2

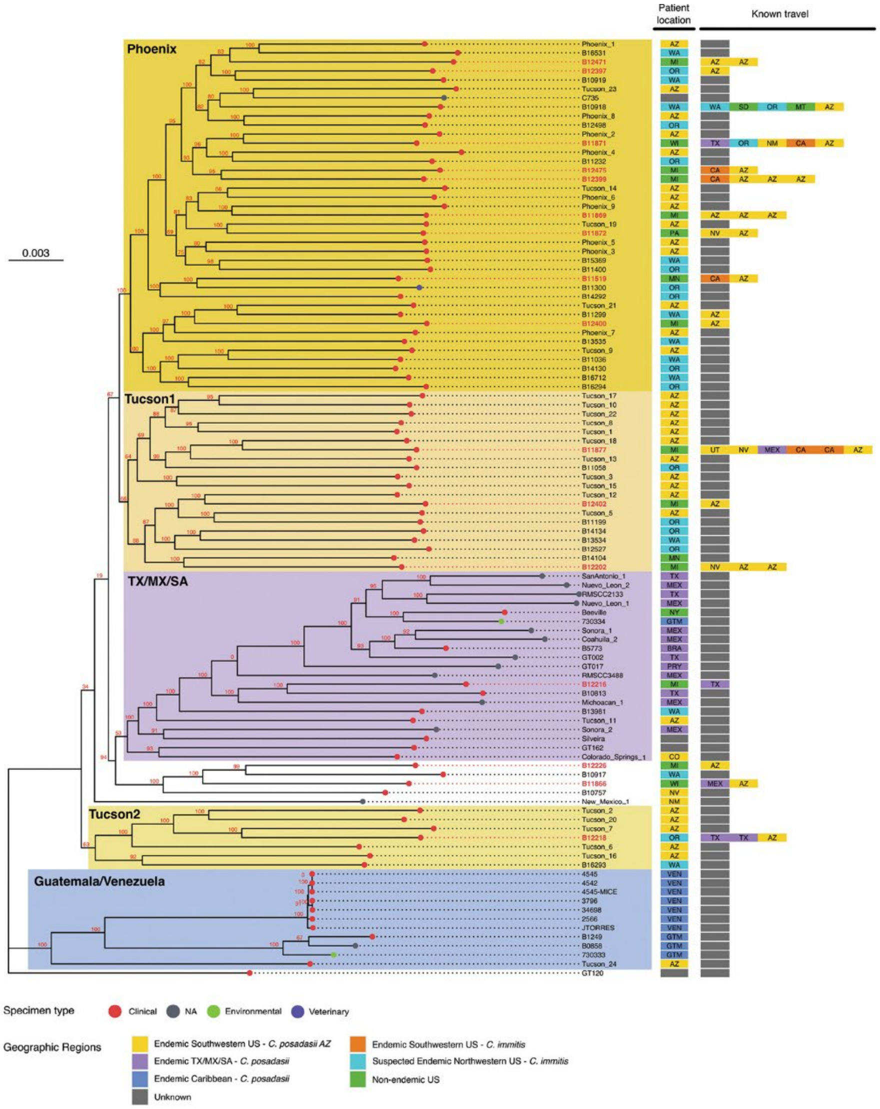


**Appendix 2 Figure 1.** Maximum likelihood phylogenetic tree for *Coccidioimycosis posadasii*. Each recognized phylogeographic clade is highlighted with a colored box labeled in its top right corner. Each leaf is labeled with locations of isolation and all known patient travel, with colors for the geographic regions. The samples sequenced for this study are shown with red bolded tip labels. Bootstrap values for each node are shown in red.

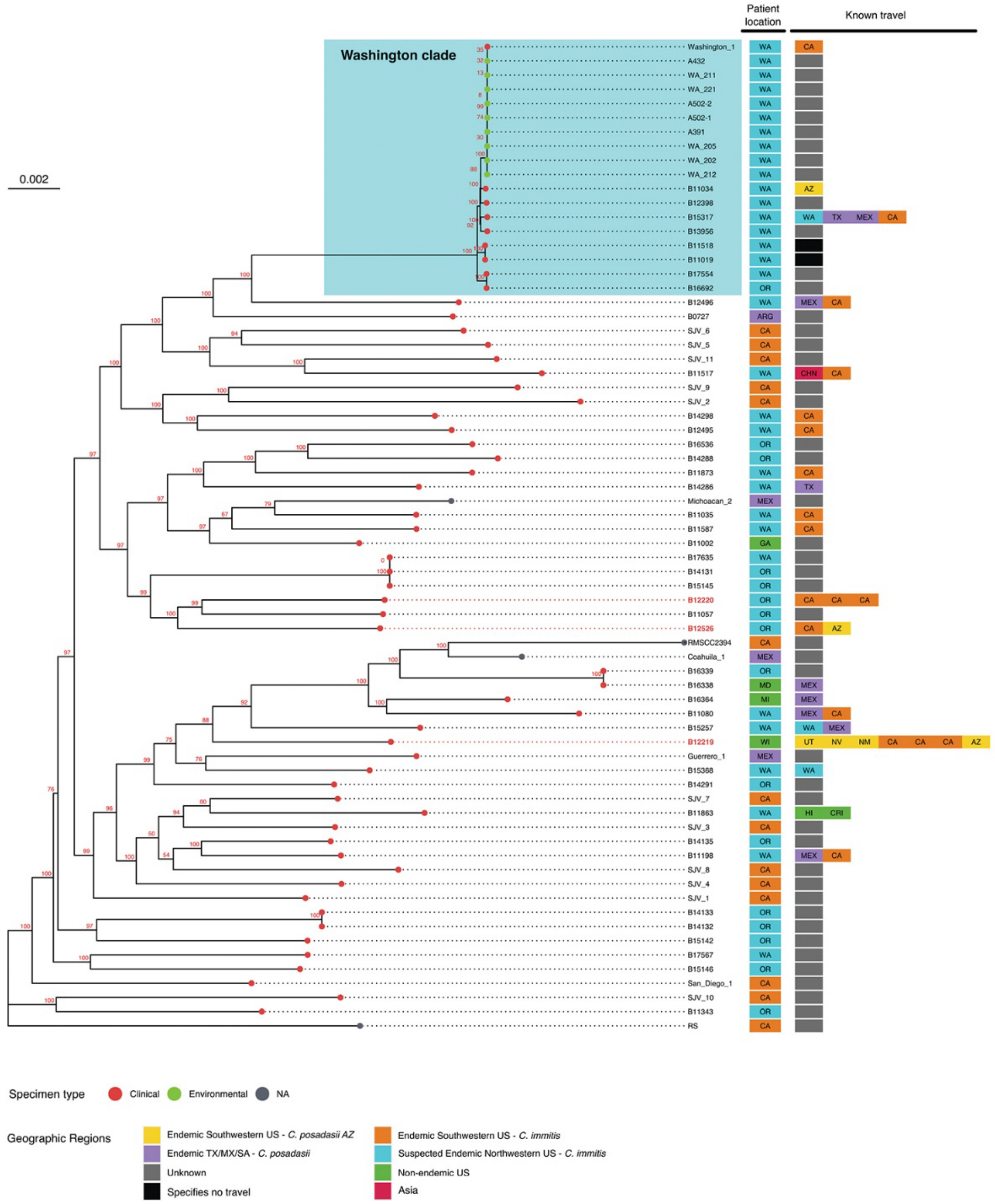


**Appendix 2 Figure 2.** Maximum likelihood phylogenetic tree for *Coccidioimycosis immitis*. Each recognized phylogeographic clade is highlighted with a colored box labeled in its top right corner. Each leaf is labeled with locations of isolation and all known patient travel, with colors for the geographic regions. The samples sequenced for this study are shown with bolded tip labels colored by the specimen type. Bootstrap values for each node are shown in red.
